# Book Notices

**Published:** 1897-02

**Authors:** 


### Book Notices.

THE JANUARY (1897) number of The Alienist and Neurologist contains "Insane Heredity. Insane and Consanguine Marriages, etc." by Dr. H. P. Stearns; "Analgesia of the Ulnar Nerve in the Insane," by Dr. Arrigo Giannone; "Report of a Case of Brain Syphilis Heroically Treated with Mercury, Followed by a Mercurial Neuritis and Recovery," by William C. Krauss, M. D.; "Interaction of Somatic and Psychic Disorder," by James G. Kiernan, M. D., Chicago; "Imperative Conceptions," a note by C. H. Hughes, M. D.; "Defence of Modern Psychiatry," by Dr. William Hirsch, New York; "Cyclone Neuroses," by C. H. Hughes, M. D., St. Louis; "On the Effects of Extirpation of the Parathyroid Glands," note by Prof. G. Vassale and Dr. F. Generali; "The Stigmata of Degeneration, a Cursory Editorial Critique," and the usual editorials, selections, reviews, book notices, etc. C. H. Hughes, M. D., is the editor, with office at 3857 Olive street, St. Louis, Mo. Subscription rates are $5 per year; single copies $1.50.

INTERNATIONAL CLINICS: A Quarterly of Clinical Lectures on Medicine, Neuralogy, Surgery, Gynecology, Obstetrics, Ophthalmology, Laryngology, Pharyngology, Rhinology, Otology, and Dermalology, and specially prepared articles on treatment. By Professors and Lecturers in the leading medical colleges of the United States, Germany, Austria, France, Great Britain, and Canada. Edited by Judson Daland, M. D. (Univ. of Penna.), Philadelphia, Instructor in Clinical Medicine and Lecturer on Physical Diagnosis in the University of Pennsylvania; Assistant Physician to the Hospital of the University of Pennsylvania; Professor of Diseases of the Chest in the Philadelphia Polyclinic, etc.; J. Mitchell Bruce, M. D., F. R. C. P., London, England, Physician to and Lecturer on the Principles and Practice of Medicine in the Charing Cross Hospital; David W. Finlay, M. D., F. R. C. P., Aberdeen, Scotland, Professor of Practice of Medicine in the University of Aberdeen; Physician to and Lecturer on Clinical Medicine in the Aberdeen Royal Infirmary, etc. Volume III. Sixth Series, 1896. J. B. Lippincott Company, Publishers, Philadelphia, Pa.

Like its predecessors, this volume contains the report of a number of clinical lectures, delivered by prominent medical men on subjects of especial interest to the practicing physician. This particular volume deals with perhaps a larger number of the more common affections, and for this reason more important ones to the physician, than we usually find in this work.

There are forty-seven lectures reported in this volume, divided as follows: On treatment, eleven; on medicine, twelve; on neurology, three; on surgery, ten; on gynecology and obstetrics, four; on ophthalmology, two; on laryngology, pharyngology, rhinology, and otology, three; on dermatology, two.

---

THE PRACTICE OF MEDICINE.—A text-book for practitioners and students with special reference to diagnosis and treatment. By James Tyson, M. D., Professor of Clinical Medicine in the University of Pennsylvania, and Physician to the Hospital of the University; Physician to the Philadelphia Hospital; Fellow of the College of Physicians of Philadelphia, etc. In one royal octavo volume of eleven hundred and eighty-four pages. Ninety illustrations. Price, in cloth, $5.50. P. Blakiston, Son & Co., Publishers, 1012 Walnut st., Philadelphia. 1896.

This book bears evidence of much labor and care in its preparation. The author announces in his preface that it represents almost purely personal work, but the book is not based on his personal practice only. To fill in the gaps of his own knowledge, he has used that of others, giving due credit.

Both the English and metric systems are used throughout the book. This, we believe, is an important matter, since the metric system will in all probability supercede the English at no distant day.

The arrangement followed by the author is a very good one, beginning with the infectious diseases, followed by diseases of the digestive system, diseases of the heart and blood-vessels, diseases of the blood and blood making organs, diseases of the thyroid gland, diseases of the urinary organs, diseases of the supra-renal capsule, constitutional diseases, diseases of the nervous system, diseases of the muscular system, the intoxications, effects of exposure to high though bearable temperature, animal parasites and the conditions caused by them, a summary of symptoms following over-doses of poisons, and the treatment of their effects, a table of minimum dose which has caused death, and maximum dose followed by recovery, and an appendix giving tables for the conversion of the English into the metric system, and the reverse.

The author's purpose to make diagnosis and treatment of disease the special features of this book has been strictly adhered to, and its practical value is very much increased thereby. It is an excellent work, and will no doubt be adopted as the text book on practice by many of the best medical schools.

S. E. H.

---

AN AMERICAN TEXT-BOOK OF APPLIED THERAPEUTICS FOR THE USE OF PRACTITIONERS AND STUDENTS. Edited by J. C. Wilson, M. D., Professor of the Practice of Medicine and of Clinical Medicine in the Jefferson Medical College; Attending Physician to the Hospital of the Jefferson Medical College, to the German Hospital, and to the Pennsylvania Hospital, Philadelphia. Assisted by Augustus A. Eshner, M. D., Professor of Clinical Medicine in the Philadelphia Polyclinic; Attending Physician to the Philadelphia Hospital. In one Royal octava volume of 1326 pages. Price, cloth, $7.00; sheep, $8.00; half morocco, $8.00; half Russia, $9.00. Sold by subscription only. W. B. Saunders, Publisher, Philadelphia. 1896.

This work, as its name implies, deals only with the treatment of disease, leaving out entirely the etiology, symptomatology, diagnosis, etc. It covers the practical part of medicine, and is just such a work as most practitioners have felt the need of.

The arrangement of the book has been based, so far as this has been possible, upon modern pathologic doctrines, beginning with the intoxications, and following with the infectious diseases due to internal animal parasites, diseases of undetermined origin, and the disorders of the digestive, respiratory, circulatory renal, nervous, and cutaneous systems.

The list of contributors number more than forty, all of them capable teachers, and all except two being Americans. Some of these have contributed articles to this work on two or more subjects, but the majority have had only one subject assigned to each of them. In this way each author has been able to give the greatest attention to the subject in hand, and the result has fully justified this arrangement. To give the reader some idea of the contributors and their subjects we mention here a few of them: Dr. I. E. Atkinson, of Baltimore, the acute poisonings, the resuscitation of persons apparently drowned, influenza; Dr. Victor C. Vaughan, of Ann Harbor, food-infection; Dr. Augustus A. Eshner, of Philadelphia, milk-sickness, miliaria—miliary fever—sweating disease, Weil's disease—infectious jaundice; Dr. Carl Frese, of Philadelphia, drug-habits; Dr. Robert T. Edes, of Jamaica Plains, Mass, chronic intoxication; Dr. John Chalmers Dabosta, of Philadelphia, septicemia and pyemia, erysipelas, anthrax, hydrophobia, tetanus, glanders, actinomycosis; Dr. W. P. Northrup, of New York City, diphtheria; Dr. Louis Starr, of Philadelphia, whooping-cough; Dr. J. W. McLaughlin, of Austin, Texas, dengue; Dr. George Dock, of Ann Arbor. the exanthemata, osteomalacia; Dr. J. C. Wilson, of Philadelphia, enteric or typhoid fever, typhus fever, relapsing fever, the plague (bubo-plague), cerebro-spinal fever, migraine; Dr. E. O. Shakespeare, of Philadelphia, cholera infectiosa (Asiatic cholera; Dr. John Guiteras, of Philadelphia, yellow fever; Dr. Beaven Rake, of London, England, leprosy; Dr. I. N. Danforth, of Chicago, croupous pneumonia, diseases of the bronchopulmonary apparatus; Dr. James T. Whittaker, of Cincinnati, tuberculosis; Dr. Orville Horwitz, of Philadelphia, syphilis; Dr. W. W. Johnston, of Washington, D. C., dysentery, diseases of the intestines—acute peritonitis; Dr. A. Laveran, of Paris, France, malarial fever; Dr. James Stewart, of Montreal, Canada, rheumatism, gout. rheumatoid arthritis, gonorrheal arthritis, rachitis, diabetes, scurvy, purpura, hemophilla; Dr. William C. Dabney, of Charlottesville, Va., diseases of the liver and of the pancreas; Dr. Wm. Osler, of Baltimore, diseases of the blood and of the ductless glands; Dr. James Tyson, of Philadelphia, diseases of the kidneys; Dr. Wharton Sinkler, of Philadelphia, neurasthenia, hysteria, the traumatic neuroses, epilepsy, infantile convulsions, laryngismus stridulus, chorea: Dr. Charles K. Mills, of Philadelphia, diseases of the brain; Dr. Henry W. Stelwagon, of Philadelphia. diseases of the skin and its appendages; Dr. Theophilus Parvin, of Philadelphia, pregnancy and its disorders. The list includes a number of others, all well known to the profession as men of eminence.

The purpose to make of this work a guide to the course of treatment to be pursued at the bedside rather than to name a list of drugs that have been used at one time or another, has been well carried out. S. E. H.

---

MEDICAL JURISPRUDENCE, FORENSIC MEDICINE AND TOXICOLOGY, by R. A. Witthaus, A. M., M. D., Professor of Chemistry, Physics and Hygiene in the University of the City of New York, etc., etc., and Tracy C. Becker, A. B., LL. B., Counsellor at Law, Professor of Criminal Law and Medical Jurisprudence in the University of Buffalo, with the collaboration of August Becker, Esq.; Chas. A. Boston, Esq.; Hon. Goodwin Brown; W. N. Bullord, M. D.; G. C. Cameron, M. D.; J. Clifton Edgar, M. D.; G. J. Edwards, Esq.; E. D. Fisher, M. D.; J. C. Johnson, M. D.; D. S. Lamb, M. D.; H. P. Loomis, M. D.; David Murray, Esq.; W. B. Outten, M. D.; Roswell Park, M. D.; W. T. Parker, M. D.; J. Parmenter, M. D.; Hon. Wm. A. Poste; Irving C. Rosse, M. D.; E. V. Stoddard, M. D.; Edward S. Wood, M. D.; George Woolsey, M. D.; J. H. Woodward, M. D. In four volumes of about nine hundred pages each. Volume IV., Toxicology. Wm. Wood & Company, Publishers, 43-45-47 East 10th st., New York. 1896.

This entire volume is devoted to the discussion of toxicology, and is from the pen of Professor Witthaus, one of the editors. After an introductory, which is really a history of toxicology from the earliest times up to the present, the author discusses, under the head of General Toxicology, the definition of poison, causation of poisoning, statistics of poisonings, absorption and distribution of poisons, methods of action of corrosives and poisons, elimination of poisons, treatment of and prognosis in poisoning, evidence in cases of murder by poison, duties of physician in cases of poisoning, the evidence of poisoning from the dead body, forensic questions, classification of poisons. Under special toxicology is discussed the corrosives, the mineral acids, sulfuric, hydrochloric, nitric and other mineral acids, fixed mineral alkalies, ammonium hydroxide, etc., the halogens. Of the mineral poisons,—antimonials, arsenical poisons, barium, bismuth, the chlorates, the chromates, copper, gold, iron, lead, mercury, phosphorus and zinc are discussed here. The vegetable poisons, first acids, acids of the acetic series, hydrocyanic acid and cyanic poisons, oxalic and tartaric acids; then the alkaloidal poisons, aconite and aconitin, the atropia group, coniin and conium and the other poisonous umbelliferous plants, gelsemium, morphia and opium, nicotin and tobacco, strychnin and nux vomica, veratrum, veratrin, etc.; next the non-alkaloidal vegetable poisons, digitalis, cocculus indicus, croton oil, lobelia, camphor, savin, tansy, etc., are all given due consideration. The poisons treated of under the head of The Animal Poisons, embrace poisonous foods, sausage, meat poisoning, poisonous fish, poisonous shell-fish, poisonous cheese, milk, etc., and cantharides.

The book closes with a chapter on the synthetic poisons, carbon monoxid, chloroform, chloral and phenol.

The list of subjects here given will give a fair idea of the contents of the volume, and when it is known that Dr. Witthaus is the author, there is little more necessary to be said; his well known reputation as a medical writer and investigator being a sufficient guarantee of the superiority of the book. One very valuable feature of this work, not indicated in the above list of the contents, is a complete bibliography, making a further investigation of the subject an easy matter. This volume completes this work, and it is now the most complete work on the subject of medical jurisprudence, forensic medicine and toxicology extant. It is really one of the great works of the nineteenth century. S. E. H.

